# Surgery or Locoregional Approaches for Hepatic Oligometastatic Pancreatic Cancer: Myth, Hope, or Reality?

**DOI:** 10.3390/cancers11081095

**Published:** 2019-08-01

**Authors:** Michele Ghidini, Angelica Petrillo, Massimiliano Salati, Shelize Khakoo, Antonio Varricchio, Gianluca Tomasello, Francesco Grossi, Fausto Petrelli

**Affiliations:** 1 Medical Oncology Unit, Fondazione IRCCS Ca’ Granda Ospedale Maggiore Policlinico, 20122 Milan, Italy; 2 Division of Medical Oncology, Department of Precision Medicine, School of Medicine, University of Campania “Luigi Vanvitelli”, 80131 Naples, Italy; 3 Department of Oncology, University Hospital of Modena and Reggio Emilia, 41125 Modena, Italy; 4 Department of Medicine, Royal Marsden Hospital, London and Surrey, Sutton SM2 5PT, UK; 5 Surgical Oncology Unit, Surgery Department, ASST Bergamo Ovest, 24047 Treviglio (BG), Italy; 6 Niguarda Cancer Center, Grande Ospedale Metropolitano Niguarda, 20162 Milan, Italy; 7 Oncology Unit, Oncology Department, ASST Bergamo Ovest, 24047 Treviglio (BG), Italy

**Keywords:** pancreatic cancer, liver metastases, oligometastatic disease, pancreatic surgery, liver resection, chemotherapy, liver metastasectomy, radiofrequency ablation, irreversible electroporation, stereotactic body radiation therapy

## Abstract

Despite extensive research, pancreatic ductal adenocarcinoma (PDAC) remains a difficult-to-treat cancer associated with poor survival. Due to the known aggressive disease biology, palliative chemotherapy is the only routinely recommended treatment in the metastatic setting in patients with adequate performance status. However, in a subset of patients with oligometastatic disease, multimodality treatment with surgery and/or locoregional approaches may provide long-term disease control and prolong survival. In fact, in highly selected cases, median overall survival has been reported to extend to 56 months in patients treated with surgery. In particular, liver and extraregional nodal resections may provide long-term tumor control with acceptable morbidity. Current guidelines do not recommend surgery for patients with metastatic PDAC and, in the case of PDAC with oligometastases, there are no published randomized controlled trials regarding locoregional or surgical approaches. Here we review the literature on surgical and locoregional approaches including radiofrequency ablation, irreversible electroporation, and stereotactic body radiation, and focus on patients with hepatic oligometastatic pancreatic cancer. We provide a summary regarding survival outcomes, morbidity and mortality and discuss selection criteria that may be useful to predict the best outcomes for such strategies.

## 1. Introduction

Pancreatic ductal adenocarcinoma (PDAC) is projected to become the second leading cause of cancer-related death by 2030 in the United States [1]. Despite extensive research in the field, prognosis remains poor in the case of advanced disease with median overall survival (mOS) ranging from 5 to 6 months for patients receiving gemcitabine monotherapy to 8–11 months for patients suitable for combination chemotherapy with nab-paclitaxel/gemcitabine or FOLFIRINOX [2,3,4].

Curative resection along with adjuvant chemotherapy offers the best possible chance of a cure. However, due to the aggressive biological behavior of PDAC, despite optimal treatment, recurrence rates remain high with 69–75% of patients experiencing a relapse within 2 years [5,6,7].

Most patients are diagnosed late, and have widespread metastatic disease at the time of presentation, which precludes curative-intent resection. However, in a small subset of patients with limited metastatic disease, intensification of multimodality approaches might result in improved outcomes and long-term disease control. The term “oligometastatic” was first proposed by Hellman and Weichselbaum in reference to an intermediary state between widespread metastatic disease and localized disease, whereby metastases are limited in number and confined to a single or limited number of organs, and where curative treatment may still be possible [8].

**Figure 1:**
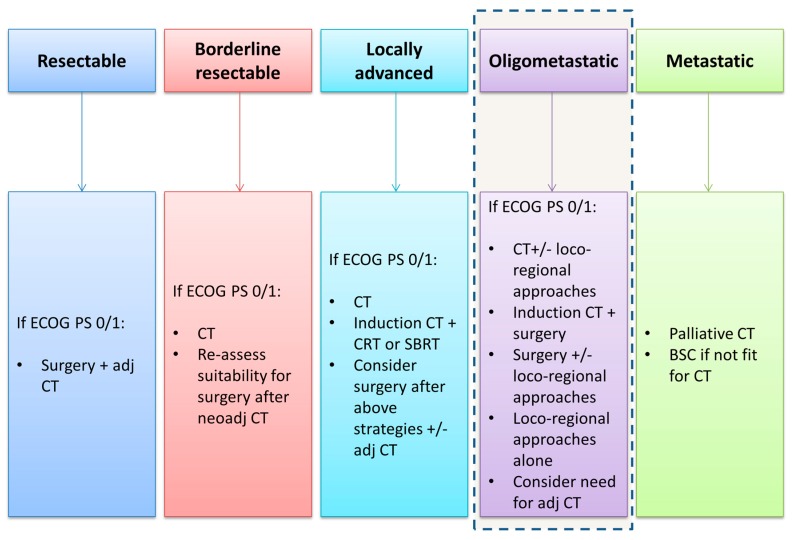
Initial treatment strategies for pancreatic cancer. Oligometastatic disease is not currently considered a separate entity in the treatment paradigm for pancreatic cancer but increasing evidence suggests that appropriately selected patients may be suitable for more intensified treatment strategies in high volume centers. ECOG PS: the Eastern Cooperative Oncology Group performance status; adj: adjuvant; CT: chemotherapy; BSC: best supportive care.

For PDAC patients with oligometastases, there has been increased interest in exploiting strategies that have successfully been used for the treatment of oligometastases in other tumor types. Such strategies include: stereotactic body radiotherapy (SBRT), metastasectomy, and radiofrequency ablation (RFA, Figure 1). Liver-directed therapies are increasingly being used to treat liver metastases from colorectal cancer in patients with technically inoperable lesions, inadequate liver remnant, and concomitant comorbidities or based on an individual’s preference. Remarkably, post-ablation 5-year OS of up to 31% has been reported for carefully selected colorectal cancer patients with liver-limited disease [9].

Apart from colorectal cancer, where liver resection is a well-established treatment in appropriately selected patients, other cancers such as gastric cancer have also been shown to derive benefit from surgical strategies with a median pooled OS of 22 months reported in a meta-analysis of gastric cancer patients proceeding with liver resection [10].

In metastatic PDAC, widespread disease, high tumor volume and poor performance status have often limited the adoption of multimodality intensive treatment strategies. However, in the locally advanced setting for tumors that do not progress after initial systemic therapy, local ablative strategies such as SBRT have already gained their place as options to improve local disease control and provide symptomatic benefit [11].

Recently, initial reports have described the feasibility and efficacy of such approaches in prolonging mOS in PDAC with oligometastases and more data are available regarding the role of metastasectomy. In particular, liver and extraregional nodal resections may enable long-term tumor control with acceptable morbidity in carefully selected patients [12].

Current guidelines do not recommend surgery for patients with metastatic PDAC [11]. However, in the case of PDAC with oligometastases, as there are no published randomized controlled trials or evidence-based guidelines regarding locoregional or surgical approaches and no consensus has been reached regarding optimal treatment strategies, we performed a systematic review of the literature aiming to evaluate morbidity and survival outcomes.

## 2. Materials and Methods

A literature search was performed to find published articles in the Medline/PubMed and Embase databases on locoregional treatment or metastasectomy for advanced/metastatic PDAC. Search terms used were (“pancreatic cancer” OR “pancreatic adenocarcinoma” OR “pancreas adenocarcinoma” OR “pancreatic ductal adenocarcinoma”) AND (metastatic OR liver metastases OR hepatic metastases OR oligometastatic OR advanced) AND (surgery OR resection OR ablation OR radiotherapy). Only studies in the English language, published between 2000 and 2019 were considered. Publications in other languages, case reports, preclinical studies, or reviews were excluded. A manual search of references of retrieved articles for additional relevant publications was performed.

## 3. Results

We identified a total of 35 studies. Among them, 10 were excluded (three reviews, five case reports, and two publications in other languages). Eventually, a total of 17 studies reporting on surgery and eight on local ablative therapies in PDAC patients with liver limited metastases were considered. The studies are discussed in the relevant sections below and summarized in Table 1; Table 2.

### 3.1. The Role of Liver Metastasectomy

Seventeen series were retrieved (Table 1) [12,13,14,15,16,17,18,19,20,21,22,23,24,25,26,27,28]; mOS ranged from 5.9 to 56 months. Survival is likely to have been influenced by the variability in inclusion criteria across studies. Seven studies reported that either all or a proportion of included patients received neoadjuvant chemotherapy [13,16,20,21,22,25,28], and one further study mentioned neoadjuvant treatment was given with no further details provided [12]. De Jong et al. authored the only study to report outcomes following liver-directed therapy strategies such as ablation in addition to resection [17]. Adjuvant chemotherapy was received by some patients in most studies [12,14,15,16,18,20,22,25,28]. In all studies, a high morbidity rate was reported, with values reaching a peak of 68% and the most frequent adverse events being pancreatic fistula (7–21%), sepsis (6–13%), and delayed gastric emptying (3–9%); mortality rate ranged between 0 and 9%. Gleisner et al. reported the worst mOS for patients with PDAC [18]. None of the patients in this study had neoadjuvant chemotherapy and only 35% of the PDAC patients had adjuvant chemotherapy. The low R0 resection rate (31.8%) may have contributed to the poor survival outcome. The best and most impressive survival outcome was reported by Frigerio et al. [16]. Perhaps this is unsurprising as all patients received neoadjuvant chemotherapy and only patients experiencing a complete disappearance of liver metastases and a decrease in cancer antigen CA 19.9 in response to chemotherapy were included. Moreover, the R0 resection rate was amongst the highest (88%). In fact if a liver lesion was still evident at surgery and cytology was positive, the procedure was abandoned. Crippa et al. demonstrated the importance of assessing response to preoperative chemotherapy to select patients for surgery [13]. Patients who had a response to chemotherapy and subsequent surgery had a significantly longer median OS of 39 vs. 11 months for patients not proceeding to resection (*p* < 0.0001). Independent predictors of OS were: primary multiagent chemotherapy, surgical resection, >5 metastases at diagnosis, and CA19-9 reduction of <50% of baseline value. However, predictors of survival have been inconsistent across studies, with Gleisner et al. reporting no factor associated with OS on univariate analysis [18], whereas Andreou at al. reported poorly differentiated cancer, R1 margin status following liver resection, no preoperative chemotherapy and no postoperative chemotherapy were predictors of a worse survival on multivariate analysis [20]. Unlike other studies, Dunschede et al. found no survival advantage for patients proceeding with synchronous liver resection compared to patients treated with chemotherapy alone (8 vs. 11 months) [15]. In fact, synchronous resections were associated with a high morbidity rate (33%). However, resection of metachronous metastases was associated with an improved mOS compared to patients treated with chemotherapy alone (31 vs. 11 months) and morbidity was reported to be 0%.

### 3.2. The Role of Radiofrequency Ablation (RFA)

Radiofrequency ablation (RFA) is based on protein denaturation with thermal coagulation caused by electrodes directly insert into the center of the tumor. Although RFA is a well-established treatment for hepatocellular carcinoma, its role in the treatment of liver metastasis from PDAC is less clear. The first study in this field was conducted by Park JB et al. in 2012 [29]. They assessed the efficacy of RFA in 34 patients with liver-only metastasis. Between 2002 and 2009, patients with ≤5 metastasis, ≤3 cm diameter in size were enrolled in the trial and underwent RFA intraoperatively at the time of pancreatic resection or percutaneously after surgery by ultrasound guidance. The authors reported a mOS of 18 months from diagnosis and 14 months from the detection of liver metastasis, including the possibility of using RFA in more than one session with good tolerability. However, although the results appear promising, it is important to note that this first attempt had no randomized controlled design and included a small sample size. A more recent Chinese retrospective analysis reported data on the safety and efficacy of RFA in 102 PDAC patients with oligometastatic disease in the liver [30]. RFA was shown to be safe and feasible and the complication rate was low (9.8%) and in line with previous reports (range: 3.54–20%) [31,32]. The most common complications that have been reported in the literature are hepatic or extrahepatic injury, pneumothorax, and hemorrhage with lower complication rates in high volume centers. However, in this study, the complications reported were vomiting, biliary-cardiac reflex, abdominal pain, and fever. The one-year survival rate was 47.1% and mOS was 11.4 months. Primary tumor location (head; mOS: 10.9 months; hazard ratio (HR): 1.868; 95% confidence interval (CI): 1.023–3.409; *p*: 0.042), size of the lesion (3–5 cm: HR: 1.801; 95% CI: 1.081–3.001; *p*:0.024), and neutrophil/lymphocyte ratio (NLR ≥ 2.5: HR: 1.716; 95% CI:1.047–2.811; *p*: 0.032) were independently associated with worse survival (Table 2).

### 3.3. The Role of Irreversible Electroporation (IRE)

Irreversible electroporation (IRE) consists of high voltage pulses, which induce cell apoptosis by creating irreversible nanopores in the cell membrane. This technique can be performed by using electrodes positioned around the tumor at the time of surgery, either laparoscopically or percutaneously, and also near major blood vessels without the typical heat sink effects induced by RFA. Generally, laparoscopic and surgical approaches are preferred due to the better visualization of primary lesions by ultrasound. Over the last few years, IRE has been evaluated for the treatment of primary pancreatic tumors, in particular, locally advanced or borderline resectable disease. Martin et al. reported the largest experience with IRE in PDAC [33]. The trial enrolled a total of 200 patients with locally advanced pancreatic cancer (stage III) to receive IRE alone or pancreatic resection plus IRE after induction chemotherapy or chemo-radiotherapy. The addition of IRE to conventional multimodality treatment showed an improvement in survival with mOS of 24.9 months (28.3 months for resection plus IRE and 23.3 for IRE alone). IRE was well tolerated, with a complication rate of 18%. On the other hand, Kluger et al. reported less favorable safety and survival outcomes in 50 consecutive patients treated with IRE. Median OS was 7.71 months with major morbidities reported as: bleeding, visceral ulcerations/perforations and portal vein thrombosis and mortality rate was high (12%) [34]. Recently the role of IRE was also investigated as part of the multimodality approach for oligometastatic disease. A single institution experience showed that the combination of chemotherapy, oligometastasectomy, and IRE to the primary tumor could represent a possible option for selected patients that demonstrate an objective response to primary chemotherapy [35]. Although this analysis showed a mOS of 16 months in 28% of patients without evident disease after the procedure, we should interpret this data with caution due to the very small sample size (seven patients) and the very high risk of selection bias (Table 2).

### 3.4. The Role of Stereotactic Body Radiation Therapy (SBRT)

Stereotactic body radiation therapy (SBRT) is a nonthermal ablative method based on the delivery of high-dose radiation from the outside to the inside of the target volume. SBRT is a well-known technique used to treat metastatic sites such as the brain and the liver as well as several primary tumors as an alternative option for patients unsuitable or unwilling to proceed with surgery (i.e., prostate [36] and NSCLC [37]). For instance, nowadays SBRT is considered for patients with metastatic colon cancer who are not candidates to receive surgery (patient’s choice, unfit, technically unresectable disease) and when other local treatments are not recommended. Although several retrospective and prospective trials have investigated the efficacy and safety of SBRT in the treatment of liver metastasis [38,39], very little is known in the context of PDAC. A small retrospective study including patients with liver-limited oligometastatic disease (eight from pancreatic cancer) treated with SBRT showed 2-year local control and OS of 85% and 38%, respectively, suggesting the feasibility of SBRT for these patients [40]. Recently, the SBRT database initiative of the German Society for Radiation Oncology (DEGRO) reviewed the data of 474 patients with oligometastatic liver disease from different cancer sites, including 5.1% with PDAC, and treated with SBRT when deemed inoperable (patient’s choice, unfit, technically unresectable disease) or not be able to receive RFA [41]. The analysis showed that only tumor volume, histology (colon versus others) and stage (early versus delayed local recurrence) were factors independently associated with OS. Most toxicities (23%) were grade 1 or 2 fatigue, nausea and diarrhea. Similar results were recently reported in the international multi-institutional RSSearch® Patient Registry experience, which included 427 patients with liver metastasis from different primary tumors (5% pancreatic) and treated with SBRT [42]. mOS and one-year survival for PDAC was six months and 18%, respectively. Although this is one of the largest observational studies reporting data for SBRT in a real-world setting, including PDAC, it is important to note the heterogeneity amongst patients, tumors and treatments within this analysis. Therefore the results cannot be generalized and only represent a large, real-life dataset. Several trials have investigated factors that could influence the prognosis of patients treated with SBRT for liver metastases. Tumor size (smaller tumor), use of high dose SBRT and histology (non-colorectal) seem to be associated with better survival (Table 2) [43,44].

## 4. Discussion

Metastatic PDAC has historically been regarded as a cancer with a very poor prognosis with survival of less than one year even with the most effective combination chemotherapy. However, a subgroup of PDAC with oligometastatic disease may experience an improvement in survival with multimodality management. In this setting, intensified treatment approaches including surgical resection of metastases and/or local ablative therapies such as SBRT, RFA, and IRE may be valid options in order to achieve disease control and long-term survival in addition to standard chemotherapy.

Despite an early meta-analysis suggesting no clear benefit for metastasectomy in PDAC [45], subsequent studies have reported encouraging survival data for patients undergoing synchronous pancreatic and liver resections [25]. In the largest series described so far on surgery for stage IV PDAC, Hackert et al. demonstrated a 5-year OS of 8.1% and 10.1% in patients undergoing resection of liver (maximum three lesions) or interaortocaval nodal metastases [12]. No differences in survival outcome were recorded based on metastatic sites, timing or administration of chemotherapy (pre- or postoperatively). Surgery was performed safely with morbidity and 30-day mortality of 45% and 2.9% for synchronous resection and 21.7% and 4.3% for metachronous liver resection, respectively.

Within a multimodality strategy, the role of systemic treatment deserves to be mentioned. In particular, a preoperative/neoadjuvant chemotherapy offers several potential advantages in this subset of patients. Indeed, it enables the early treatment of micrometastatic disease and allows for downsizing of tumors with a subsequent increase in the potential for an R0 resection. Additionally, it can provide some insight into disease biology by sparing refractory patients or those progressing on chemotherapy from unnecessary surgery.

In line with this, Frigerio and colleagues reported on a highly selected subset of PDAC patients with oligometastases that achieved impressive survival outcomes after neoadjuvant chemotherapy and surgery [16]. The authors only included patients with liver-limited disease who experienced sustained complete radiological response to preoperative chemotherapy and normalization or significant reduction in CA19.9. Amongst them, 88% had an R0 resection, 17% had a complete pathological response, and median disease-free survival and mOS were 27 and 56 months respectively. Notably, the mortality rate was 0% and grade B/C pancreatic fistula occurred in 16%/4% of cases.

It is also worth considering which chemotherapy regimen is optimal as an induction treatment. Following chemotherapy with FOLFIRINOX, 60% of borderline resectable, and 20–25% of unresectable PDAC were able to receive radical surgery which suggests that it is an appropriate option in this setting [46]. Timing of the appearance of metastases (synchronous vs. metachronous) is another important factor in the decision making process for proceeding to surgery. While a small case series suggested a potential survival advantage for liver resection in metachronous liver deposits from PDAC compared to chemotherapy alone (mOS 31 vs. 11 months) [15], this has not been replicated in other larger cohorts. Although a long disease-free interval after pancreatic surgery may reflect a more favorable biological behavior in patients with metachronous liver disease, the initial presence of hepatic lesions should not represent an absolute contraindication to surgery.

More recently, there has been increased interest in the role of intraoperative radiotherapy (IORT). This is primarily due to the retroperitoneal location of the pancreas and its close proximity to other organs such as the liver and intestines which make it more difficult to deliver effective radiotherapy doses externally. IORT has been associated with decreased local recurrence rates in patients suitable for resection of their primary and a median survival time of 19.1 months [47]. In a study of 194 patients with locally advanced pancreatic where intraoperative radiotherapy was delivered, the median survival was 12 months and local control at 2 years was 41% [48]. Similar results were achieved in another study of locally advanced, unresectable pancreatic cancer where 6-month and 12-month survival rates were 100% and 57.1%, respectively, for patients treated with intraoperative radiotherapy compared to 42.9% and 0% for patients treated with palliative therapy [49]. Given the promising results in the locally advanced setting, it is possible that intraoperative radiotherapy may become a strategy worth considering for patients with oligometastatic disease that are suitable for intensified treatment approaches. Notably, the most challenging concept is the appropriate selection of patients for more aggressive treatments. Well-established clinical factors such as age, performance status, the likelihood of achieving R0 resection, low metastatic burden, liver, and/or paraaortic lymph nodes involvement may help in the decision making process. However, novel and more robust biomarkers are needed to improve the accuracy of treatment selection. Tumor markers such as CA19-9 have been shown to predict resectability and early recurrence after surgical resection in earlier stage disease. In fact, elevation of CA 19-9 has been reported to be associated with decreased stage-specific survival, with the greatest difference being between stage I and II (HR 1.26, *p* < 0.001) [50]. The prognostic/predictive value of tumor markers remains to be ascertained in this patient population.

Recently, molecular subtyping of PDAC through multiplatform analysis has unveiled distinct subgroups that are associated with prognosis and response to treatment [51]. Subtyping cancer on the basis of clinically and biologically relevant molecular similarities and differences might enable opportunities to improve risk-stratification, optimize individual patient management, and improve overall outcome. To this end, in the Comprehensive Molecular Characterization of Advanced Pancreatic Ductal Adenocarcinoma for Better Treatment Selection (COMPASS trial NCT02750657) [52], the basal-like subtype has been associated with reduced chance of response to first-line chemotherapy. Thus, in the context of limited-metastatic disease, we can speculate that basal-like tumors may benefit the most from an aggressive surgical approach rather than systemic treatment. Similarly, a recent study recognized that there are long-term survivors (>18 months) amongst metastatic PDAC patients, showing that a low neutrophil-to-lymphocyte ratio was associated with long-term survival. The incorporation of the neutrophil-to-lymphocyte ratio could therefore be used as a stratification factor for more intensive multimodality treatments [53]. It has to be acknowledged that all the literature available today on this topic to date includes mainly small, uncontrolled, retrospective studies, thus precluding any definitive conclusions. Therefore, larger randomized-controlled trials are required to assess the feasibility and efficacy of such intensified multimodality strategies. Following the example of the RENAISSANCE AIO-FLOT-5 trial (NCT02578368) in gastric cancer [54], a future goal might be to compare conversion chemotherapy plus surgery/local therapies against standard first-line chemotherapy in PDAC patients with oligometastases.

## 5. Conclusions

Advanced PDAC remains a disease that is challenging to treat with an absolute improvement in median OS of a few months with palliative chemotherapy in the last decade. Metastasectomy and ablative therapies are increasingly being considered for PDAC patients with limited metastatic disease. Currently, there is inconsistent evidence to recommend such strategies in everyday clinical practice as current guidelines do not recommend surgery for patients with metastatic PDAC and there are no published randomized controlled trials regarding locoregional or surgical approaches. However, in high-volume specialized centers they could be considered on an individual basis and after multidisciplinary discussion, since highly selected cases might achieve long-term disease control with more aggressive approaches. Appropriate patient selection for such strategies remains a challenge, as studies have not consistently identified a group of patients that will definitely benefit. Patients with a low number of liver limited metastases, metachronous or synchronous metastases that show a response to neoadjuvant chemotherapy, appear to be the candidates that are likely to benefit the most from intensified treatment strategies. Future prospective trials are warranted to fully address the potential role of metastasectomy and ablative therapies in patients with oligometastatic PDAC.

## Figures and Tables

**Table 1:** Studies evaluating liver metastasectomy in pancreatic cancer.

Name (Year) Country	*N*	Patient Characteristics	Treatment in Addition to Surgery	Surgery Details	Morbidity Rate %	Complication Type (%)	Mortality Rate %	Median OS Months
Hackert [12] (2016) Germany	128	- mean age 61 - 48% male - res of liver (*n* = 85) or ILN mets (*n* = 43)	- neo-adj treatment given *n* = 20, (16%) - adj CT completed *n* = 73 (57%), incomplete in *n* = 22 (17%) and unk for *n* = 33 (26%)	- ILN + sync pancreatic res (*n* = 43) - 72.9% primary + sync liver res - 22.4% meta liver res (mean of 18.4 months after primary)	- 45 for sync res - 22 for meta res	wound infection *n* = 14 (11), percutaneous drainage *n* = 11 (9), delayed gastric emptying *n* = 11 (9)	- 3 for sync res - 4 for meta res	- 12.3 from liver res sync or meta - 12.3 for ILN res
Crippa [13] (2016) Italy	4	- median age 65- 60% male - all treated with CT - res of primary only (*n* = 7) - sync liver res (*n* = 3) - meta liver res (*n* = 1) - 1 lesion (*n* = 3) - 2 lesions (*n* = 1)	pre-op CT for the 11 res pts - FOLFIRINOX *n* = 3 - GEMOX *n* = 2 - PEXG *n* = 4 - PDXG *n* = 1 - PEFG *n* = 1	- res: median 12 months from diagnosis (range 6–20 months) - sync liver res (*n* = 3) - meta liver res (*n* = 1)	- 27	- fistula, *n* = 2 (18) - post-op pneumonia, *n* = 1 (9)	- 0	- 39 for res pts (*n* = 11) vs. 11 without res(*n* = 116), *p* < 0.0001 - 39 for pts proceeding to res (*n* = 11) vs. 12 for pts with partial response to CT managed without res (*n* = 45), *p* < 0.0001 - 11 for whole population (*n* = 127)
Klein [14] (2012) Germany	22	- mean age 58 - male 64% - incidental liver mets at surgery	-adj CT with gemcitabine in all pts	- Sync liver res in all pts	- 18	- fistula, *n* = 2 (9) - hemorrhage, *n* = 2 (9)	- 0	- 7.5 in pts having sync liver res (*n* = 22) vs. 14.4 in control group having res of primary alone (*n* = 22), *p* = 0.15
Dunschede [15] (2010) Germany	13	sync liver res pts - mean age 55 - 56% male - median number of liver mets = 3 (range 1–5) meta liver res pts - mean age 42–50% male - median number of liver mets = 1.75 (1–2)	- 2 pts had adj CT after primary res	- sync liver res pts (*n* = 9) - meta liver res pts (*n* = 4)	- 33 for sync res - 0 for meta res	NA	- 0 for sync res - 0 for meta res	- 8 in sync res group (*n* = 9) vs. 11 in the CT alone group (*n* = 10) - 31 from detection of mets in meta res group (*n* = 4) vs. 11 in the CT alone group (*n* = 10)
Frigerio [16] (2017) Italy	24	- mean age 58 - male 46% - number of liver mets at baseline: 1, *n* = 5 2, *n* = 5 multiple *n* = 14	pre-op CT in all pts: - gemcitabine *n* = 5 - FOLFIRINOX *n* = 16 - gemcitabine + nab-paclitaxel *n* = 3 - adj CT in 15/24 (63%)	- surgery only for pts with downstaging after CT with disappearance of liver lesions - If liver lesion evident at surgery, fine needle aspiration and surgery aborted if positive - R0 res in 21/24 (88%)	- 63	- grade B/C -fistula *n* = 5 (21), - bleeding *n* = 1 (4) - sepsis *n* = 3 (13)	- 0	- 56
De Jong [17] (2010) USA	126	- PDAC *n* = 42 - others *n* = 84 - median age 56- male 59% - median number of treated liver mets = 2 (range 1–15)	- res only *n* = 57 (45%) - res + ablation *n* = 14 (11%) - ablation only *n* = 10 (8%) - TACE only *n* = 6 (5%) - whole liver RT *n* = 28 (22%) - unk *n* = 11 (9%)	- sync liver-directed therapy with primary res *n* = 57 - staged primary res and liver -directed therapy *n* = 69	34 overall - sync *n* = 15 (26) - staged *n* = 28 (41)	- liver abscess *n* = 14 (11) - surgical site infection *n* = 11 (9) - sepsis *n* = 8 (6)	2 overall - sync *n* = 1 (2) - staged *n* = 2 (3)	- 17.7 for PDAC + liver-directed therapy - 20.1 for whole population
Gleisner [18] (2007) USA	22	- mean age 65 - 46% male - PDAC *n* = 17 - others *n* = 5	6 pts had adj CT - 5-fluorourcil (*n* = 3) - gemcitabine (*n* = 3)	- all sync liver res (*n* = 22)	46	- urinary retention *n* = 2 (9) - delayed gastric emptying *n* = 2 (9) - fistula *n* = 2 (9)	9	- 5.9 for PDAC vs. 9.9 for non-PDAC, *p* = 0.43
Adam [19] (2006) France	40	- PDAC *n* = 40 - mean age for all pts = 53 (multiple primary tumors)	NA	NA	NA	NA	NA	for PDAC: - 20 - 25% 5 year survival rate
Andreou [20] (2018) Germany	76	- median age 64 - 60% male - 36% multiple liver mets - median number of mets = 1 (range 1–5)	- pre-op CT *n* = 4, (5%) - adj CT *n* = 55, (72%)	- all sync liver res (*n* = 76)	50	- fistula *n* = 13 (17) - hemorrhage *n* = 5 (7)	5	survival rates: - 41% 1 year - 13% 3 year - 7% 5 year
Wright [21] (2016) USA	11	- 23 pts had primary res - mean age 58 - 61% male - 11/23 had metastasectomy - liver res *n* = 9 - lung res *n* = 2	pre-op CT in all pts: - FOLFIRINOX *n* = 14 (61%) - gemcitabine based *n* = 9 (39%)	- median time from diagnosis to surgery 9.7 months for all 23 pts	- 13	NA	- 0	- 18.2 from time of surgery survival rates: - 72.7% 1 year - 21.5% 3 year
Kandel [22] (2018) USA	6	- median age 64 - 100% male	- pre-op CT *n* = 5 - pre-op CT + RT *n* = 1 - adj CT *n* = 5 - adj CT + RT *n* = 1	- sync liver res *n* = 1 - sync liver res + RFA to liver *n* = 2 - radio-embolization to liver only *n* = 1 - RFA only for lung mets *n* = 2	NA	NA	NA	- 33 for surgery (*n* = 6) vs. 11.8 in patients with M1 disease and no surgery (*n* = 18), *p* = 0.01
Bahra [23] (2015) Germany	21	- median age 60 - 58% male - 21 had liver res	- all pts had gemcitabine based adj CT	- sync liver res (*n* = 21)	20	- fistula grade C *n* = 3 (7) - hemorrhage *N* = 2 (4)	2	- 10.4 for cytoreductive surgery + gemcitabine based CT vs. 7.2 for CT alone *p* = 0.009
Zanini [24] (2015) Italy	15	- median age 55 - 53% male - single lesions *n* = 9 (60%) - multiple lesions *n* = 6 (30%)	- all pts had gemcitabine based adj CT	- 11 had sync liver res - 4 had meta liver res. - median disease-free interval between primary surgery and diagnosis of metastases 8 months	60	- fistula grade B/C *n* = 2 (13)	0	- 9.1 -11.4 for meta vs. 8.3 for sync, *p* = 0.038
Tachezy [25] (2016) 6 European pancreas centres	69	- median age 65 - 57% male - median number of mets = 2 (range 1–11)	- no pre -op CT *n* = 59 pre-op CT: - gemcitabine *n* = 3 - FOLFIRINOX *n* = 4 - unk *n* = 2 - RFA *n* = 1 - Adj CT: - gemcitabine *n* = 35 - FOLFIRINOX *n* = 3 - other CT *n* = 5 - none *n* = 11	- 69 sync liver res	68	- fistula grade B/C *n* = 9 (13) - wound infection *n* = 12 (17) - hemorrhage *n* = 6 (9)	1%	- 14.5 for sync res vs. 7.5 for a control group having no res *p* < 0.001
Slotta [26] (2014) Germany	13	- multiple different primary tumor types, PDAC *n* = 13 - mean age for whole population = 59 (multiple primary tumors) and 44% male	NA	NA	NA	NA	NA	- 8.2 for PDAC
Schiergens [27] (2016) Germany	19	- multiple different primary tumor types, PDAC *n* = 13 - mean age for the 43 pts with gastrointestinal tumors = 64, 53% male	NA	NA	NA	NA	NA	- 7 for PDAC
Shrikhande [28] (2007) Germany	29	- median age 65 - 38% male - incidental liver mets discovered at surgery *n* = 14 - liver mets discovered after surgery *n* = 15	- neoadjuvant CT + RT *n* = 1 - adj CT *n* = 23 gemcitabine *n* = 13 fluorouracil *n* = 6 experimental tumor vaccine *n* = 2 other CT *n* = 2	- liver res *n* = 10 - ILN res *n* = 10 - peritoneal *n* = 8 - ILN+liver res *n* = 1	24%	- fistula *n* = 2 (7) - delayed gastric emptying *n* = 1 (3) - intra-abdominal abscess *n* = 2 (7) - hemorrhage + delayed gastric emptying *n* = 1 (3)	0	- 13.8 - 1 year survival rate of 58.9% - 27 for ILN mets - 11.4 for liver mets - 12.9 for peritoneal mets

Legend: adj: adjuvant; CT: chemotherapy; *n*: number of patients intended for metastasectomy; ILN: interaortocaval nodal; PDAC: pancreatic ductal adenocarcinoma; meta: metachronous; mets: metastases; sync: synchronous; OS: overall survival; pts: patients; post-op: postoperative; RT: radiotherapy; FOLFIRINOX: oxaliplatin, irinotecan, fluorouracil, and leucovorin; PEXG/PDXG: cisplatin, capecitabine, gemcitabine + either epirubicin (PEXG) or docetaxel (PDXG); PEFG: cisplatin, epirubicin, fluorouracil, and gemcitabine; res: resection/resected; TACE: trans-arterial chemo-embolisation; unk: unknown.

**Table 2:** Studies evaluating local ablative therapies for pancreatic ductal adenocarcinoma (PDAC) with oligometastases.

Name(Year)	*N*	Design	Histology % Metastatic Site %	Preop CT/ RT Yes/No (%)	Type of Procedure %	Morbidity Rate %	Complication Type %	mPFS Mo	mOS Mo
Park [29] (2012) Korea	34	retro	- PDAC 100 - liver mets 100	no (100)	- intraop orpostop RFA 100 - surgery 100	NA	- pleural effusion 8.8 - liver abscess 3	2 (range: 0–32)	18 14 (OS after liver mets)
Hua [30] (2017) China	102	retro	- PDAC 100 - liver mets 100	no (100)	RFA 100	9.8	- vomiting 2 - biliary-cardiac reflex 2 - abdominal pain 4 - fever 2	NA	11.4
Martin [33] (2015) USA	200	pros	- PDAC 100 - locally advanced 100	CT (100) CT + RT (52)	- IRE alone (75) - pancreatic res plus IRE (25)	18 mortality 1.5	- gastrointestinal 23 - liver 10 - infection 9 - vascular 5.5	12	24.9
Hong [35] (2018) USA	7	retro	- PDAC 100 - liver 57.1 - omentum 42.8 - peritoneum 42.8	CT (100)	- IRE plus resection 86 - IRE on mets 14.3	NA	NA	NA	16
Kluger [34] (2016) USA	50	pros	- PDAC 94 - neuroen 6	CT (94) RT (83)	IRE 100	38 mortality 11	NA	NA	12
Yuan [40] (2014) China	57	retro	PDAC 14	NA	SBRT 100	fatigue, nausea, vomiting, and changes in liver function tests	NA	NA	2-year OS: 38
Andratschke [41] (2018) Switzerland	474	retro	- PDAC 5.1 - liver 100	NA for PDAC	SBRT 100	fatigue, nausea, and diarrhea	Hepatitis 0.4 Liver fibrosis 1.4 Necrotic reaction 0.4	NA	NA for PDAC
Mahadevan [42] (2018) USA	427	retro	- PDAC 4.9 - liver 100	NA for PDAC	SBRT 100	NA	NA	NA	6 1-year OS 18%

Legend: CT: chemotherapy; IRE: irreversible electroporation; NA: not available; neuroend: neuroendocrine tumor; No: number; OS: overall survival; PDAC: pancreatic ductal adenocarcinoma; pros: prospective; retro: retrospective; RFA: radiofrequency ablation; RT: radiotherapy; SBRT: Stereotactic body radiation therapy.
